# Informed Consent Practices for Hip Fracture Surgeries at a Tertiary Care Hospital in Wad Madani, Sudan

**DOI:** 10.7759/cureus.65043

**Published:** 2024-07-21

**Authors:** Ahmed Mohamed, MohammedElhassan Abdalla

**Affiliations:** 1 Department of Orthopaedics, Gezira Centre for Orthopedic Surgery and Traumatology, Wad Madani, SDN

**Keywords:** wad madani, consent practices, royal college of surgeons of england, general medical council, consent, fractures, neck of femur

## Abstract

Introduction: An essential component of medical ethics and practice is informed consent. The General Medical Council (GMC) and the Royal College of Surgeons of England (RCS) provide guidelines for obtaining valid consent. Failing to obtain sufficient or valid consent can have legal consequences.

Materials and methods: Over a period of two and a half months, from March 12 to May 28, 2022, a retrospective cross-sectional study was conducted to evaluate consenting practices for neck of femur fracture surgeries. A total of 88 patient consent forms were reviewed. The standard consent forms utilized in this study were those endorsed by the British Orthopaedics Association (BOA) and were based on the guidelines provided by the RCS and the GMC.

Results: Resident surgical trainees and medical officers obtained the majority of the consents, 31 (35.22%) and 49 (55.68%), respectively. The most frequently reported risks included infection, blood clots (deep vein thrombosis and pulmonary embolism), bleeding, and wound complications. Neurovascular injury was not mentioned in 75 (85.33%) consent forms. Additionally, hip stiffness, prosthetic dislocation, death, and leg length discrepancy were not discussed with any of the patients. Additionally, we observed that the diagnosis or reason for surgery was mentioned in only 60 (68.18%) consent forms. Furthermore, none of the forms specified the intended benefits, the necessity for a blood transfusion, or the patient identification details.

Conclusion: Our study revealed inadequate documentation of surgical risks in patient consent forms for neck of femur fracture surgeries, with orthopaedic-specific risks often overlooked. This issue likely results from insufficient orthopaedic training among the medical officers and junior resident trainees responsible for obtaining consent. We recommend induction teaching sessions to improve their understanding of standard consenting practices and associated risks, along with implementing patient identification stickers.

## Introduction

Informed consent is a fundamental principle in medical ethics and practice. It ensures that patients are fully aware of the potential benefits, risks, and alternatives of a proposed treatment or procedure before they agree to it [1]. This process not only respects the patient's right to autonomy but also helps build trust between the patient and the healthcare provider. It's crucial for physicians to communicate all relevant information clearly and comprehensively to enable patients can make decisions that align with their values and preferences [1].

Junior medical staff members frequently obtain informed consent for elective surgeries during pre-assessment clinics or on the day of the procedure. House officers (HOs) and medical officers often fall into this category. In the United Arab Emirates (UAE), according to the guidelines set forth by the Department of Health (DOH), it is the primary responsibility of the attending physician or their designated representative to ensure that the patient's informed consent is obtained prior to undertaking any surgical, dental, diagnostic, or invasive procedures [2].

A concise explanation of the intended procedure, including the type of anaesthetic used, should be provided before consent is obtained. Medical jargon should be avoided, as it can make the information more difficult to understand. The explanation should include sufficient information for decision-making, covering (i) the risks and benefits involved, (ii) any alternative therapies, and (iii) the risks and advantages of opting for no treatment.

Comprehensive guidelines for what constitutes valid consent are provided by the General Medical Council (GMC) [3] and the Royal College of Surgeons of England (RCS) [4]. For consent to be considered valid, the patient must have the capacity to make decisions, provide their consent voluntarily, and have access to sufficient and relevant information. Legally speaking, it is crucial to have a thorough conversation with the patient about the planned surgery and to ensure that all information is documented in writing.

In Wad Madani, Sudan, the Gezira Centre for Orthopaedic Surgery and Traumatology is a prominent tertiary hospital that caters to both the local population and the surrounding villages. This department is staffed by a diverse team of medical professionals, including house officers, resident trainees, specialists, and consultants, who collaborate to provide comprehensive orthopaedic care. The department is well-equipped to perform numerous elective orthopaedic procedures, with a daily trauma surgical list.

It is not surprising that neck of femur (NOF) fractures are particularly prevalent among the elderly; these fractures are predominantly caused by low-energy trauma, which is more likely to occur in individuals with osteoporotic bone [5]. Hip fractures are commonly treated in our facility with hemiarthroplasty and internal fixation using an intramedullary nail, dynamic hip screw, or dynamic condylar screw. Unfortunately, our centre does not perform total hip replacements.

To prepare patients for surgical management of their NOF fractures in accordance with the GMC and the RCS guidelines, the study aimed to assess the quality and accuracy of documentation regarding surgery-associated risks during patient consent.

## Materials and methods

This was a retrospective cross-sectional assessment of the consenting procedures for neck fracture surgeries carried out between March 12 and May 28, 2022, at the Gezira Centre for Orthopaedics and Traumatology, Wad Madani, Sudan. 

In Sudan, it is traditional for a first-degree relative to provide consent for procedures, even if the patient is fully capable. Thus, the consent forms were found to have been signed by either the patient or a first-degree relative. Nevertheless, the patient's full capacity to consent was not indicated in those signed by a relative.

The inclusion criteria were patients who were diagnosed with NOF fractures and underwent surgical treatment during the study period. The study was restricted to adult patients aged 55 years and older. The exclusion criteria were designed to ensure that the study remained relevant and accurate. Patients who were diagnosed with NOF fractures but did not undergo surgery were excluded, as were those with missing consent forms. The study did not include any patients who underwent surgery outside the specified study period, either before March 12, 2022, or after May 28, 2022. Additionally, in order to preserve the study's focus, patients with fractures in locations other than the femur neck were excluded. The study also excluded patients under the age of 55 and those who had initially provided consent but subsequently revoked it.

Despite advancements in digital technology, our facility continues to rely on paper forms due to the lack of an integrated computer system. The office where all patient files are kept after discharge provided the information about the number of patients with NOF fractures who were admitted as well as those who had surgery during the study period. 

We used the following sources as the standards for our study: the RCS Consent: Supported Decision-Making (2016) [4], the GMC Guidance on Professional Standards and Ethics for Doctors: Decision Making and Consent (2020) [3], and the model consent form for hip fracture surgeries available on the British Orthopaedic Association website [6]. Each consent form was then examined separately to determine whether the documentation complied with the GMC and RCS guidelines. IBM SPSS Statistics for Windows, Version 25.0 (Released 2017; IBM Corp., Armonk, New York, United States) was used to perform the statistical analysis.

## Results

A total of 88 consent forms for neck fracture surgeries were included in the study. Of these, 51 (57.95%) underwent hip hemiarthroplasty, while 37 (42.04%) underwent surgical fixation with either a dynamic hip screw, dynamic condylar screw, or an intramedullary nail. Consent for the surgery was obtained by an MO without prior formal orthopaedic training in 49 (55.68%) cases, and by a resident in 31 (35.22%) cases. A specialist obtained consent in six (6.82%) cases, while a consultant obtained consent from only two individuals (Table 1).

**Table 1:** Summary of the surgical procedures performed for neck of femur fractures and the individual obtaining consent IMN: intramedullary nail; DCS: dynamic condylar screw; DHS: dynamic hip screw

Type of Surgery	Number	Percentage
Hemiarthroplasty	51	57.95
Internal hip fixation (IMN/DHS/DCS)	37	42.04
Person taking the consent		
Consultant	2	2.27
Specialist	6	6.82
Resident trainee	31	35.22
Medical officer	49	55.68

Infection, blood clots (deep vein thrombosis and pulmonary embolism), and bleeding were the most commonly reported risks with 80 (90.90%), 70 (79.55%), and 77 (87.50%) consent forms, respectively. Additionally, wound-related complications occurred at a rate of 83 (94.32%) forms. It's interesting to note that none of the patients received information about the possibility of developing joint stiffness in the future, leg length discrepancies, joint dislocation, or death. Table 2 presents a summary of the documentation rates for the risks associated with surgery.

**Table 2:** An overview of the rates of documentation for surgical risks in the reviewed consent forms (N=88)

Risks	Number of consent forms reporting the risk	Percentage
Infection	80	90.90
Leg length discrepancy	0	0
Blood clots (deep vein thrombosis, pulmonary embolism)	70	79.55
Bleeding	77	87.50
Neuro-vascular injury	13	14.77
Prosthetic dislocation	0	0
Postoperative pain	8	9.10
Failure/malunion/non-union/loosening of prosthesis	28	35
Non-union/Malunion	32	36.36
Death	0	0
Wound-related complications	83	94.32
Hip stiffness	0	0

Following a more thorough examination of the 88 consent forms, it was determined that 84 (95.45%) accurately indicated the side of the surgery, while 86 (97.72%) accurately listed the procedure's title. Nevertheless, the diagnosis was included in only 60 (68.18%) forms. It is important to note that none of the forms included information regarding the potential necessity for a blood transfusion, the intended benefits, or the patient identification details. Furthermore, the type of anaesthesia to be administered was not specified in 12 (13.64%) forms. The consenting physician's details, which included their name, title, signature, and date of signing, were fully completed in 80 (90.90%) of the consent forms. Table 3 shows an overview of the completeness of the remaining elements of the consent form.

**Table 3:** An overview of the completeness of the remaining elements of the consent forms (N=88)

Component assessed	Number of forms with complete details	Percentage
Title of the surgery	86	97.72
The side of the injury and surgery	84	95.45
Patient identification details (name, Date of birth, sex, hospital number)	0	0
Consenting doctor details (name, title, signature, and date)	80	90.90
Intended benefits	0	0
Diagnosis	60	68.18
Type of anaesthesia	66	75
Probable need for blood transfusion	0	0
Responsible Consultant name and job title	61	69.31
Signature	83	94.31
Patient/relative name and date of signing	83	94.31

## Discussion

Fractures of the NOF remain a significant global public health concern. As the population ages worldwide, the incidence of these fractures is expected to increase, along with rising healthcare costs and expenses [7]. It is not surprising that NOF fractures are more common in the elderly population, as they are typically the result of low-energy trauma in osteoporotic bone [5].

In the 2015 case of Montgomery v. Lanarkshire Health Board, a Supreme Court ruling significantly changed the law on informed consent [8]. It now mandates that physicians must take reasonable care to ensure that the patient is aware of any material risks involved in any recommended treatment, as well as of any reasonable alternative or variant treatments.

The RCS and the GMC mandate that informed consent must be obtained prior to any surgical procedure [9,10]. While the ultimate responsibility for obtaining consent lies with the healthcare professional proposing and performing the procedure, this task can be delegated to an individual who has received appropriate training and possesses specific knowledge about the procedure and its associated risks [9]. Regrettably, in many hospitals, this responsibility is often assigned to the least experienced team member. These individuals may lack comprehensive knowledge of all potential risks due to their limited experience [11,12]. Consequently, this practice can result in significant gaps and insufficiencies in the consent process [13,14]. This underscores the critical importance of established guidelines and standards.

Our centre, and potentially the entire nation, lacks access to labels that contain patient identification information, which is why none of the forms displayed patient identification details. Additionally, a significant number of patients with hip fractures may require blood transfusions due to blood loss during the procedure or pre-existing anaemia. However, in our investigation, we found that none of the cases indicated the potential need for a blood transfusion. Nonetheless, we routinely prepare two units of blood before the surgery. Generally, patients value informed consent and expect to receive all pertinent information [15].

In our investigation, we observed that general operative risks typical in orthopaedic surgeries, such as infection, bleeding, blood clots, and general wound complications, were consistently documented. This finding aligns with Perera et al.'s study [1], which similarly identifies these risks, with the addition of postoperative pain and neurovascular injuries, but excludes specific wound-related complications.

Singh et al. conducted an audit in which they reviewed 26 consent forms for all adult patients with capacity who were undergoing surgical repair of traumatic hip fractures in both the first and second cycles [16]. They concluded that targeted teaching on consenting practices yields the greatest benefits.

Our study has several limitations. Firstly, it was conducted at a single centre, which may limit the generalizability of the findings to other settings and patient populations. Additionally, the study sample was relatively small, which could affect the statistical power and robustness of our results. Furthermore, a limitation is that we did not take into consideration potential complications related to anaesthesia.

## Conclusions

Our study identified deficiencies in patient consent for NOF fracture surgeries, with insufficient documentation of surgical risks. Orthopaedic-specific risks were less frequently mentioned compared to general surgical risks, possibly due to inadequate training among MOs and junior resident trainees responsible for consent.

To address these issues, we recommend conducting a teaching session during the induction of each new batch of senior HOs and junior resident trainees. This session should focus on standard consenting practices and enhance their understanding of hip fractures, surgical management, and potential risks. Additionally, patient identification stickers should be implemented
